# A portable low-cost device to quantify advective gas fluxes from mofettes into the lower atmosphere: First application to Starzach mofettes (Germany)

**DOI:** 10.1007/s10661-023-12114-8

**Published:** 2024-01-11

**Authors:** Yann Georg Büchau, Carsten Leven, Jens Bange

**Affiliations:** Center for Applied Geoscience, University of Tübingen, Schnarrenbergstr. 94-96, Tübingen, 72076 Baden-Württemberg Germany

**Keywords:** $\text {CO}_{2}$, Degassing, Earth mantle, Low-cost, Monitoring

## Abstract

In this study, we introduce a portable low-cost device for in situ gas emission measurement from focused point sources of CO_2_, such as mofettes. We assess the individual sensors’ precision with calibration experiments and perform an independent verification of the system’s ability to measure gas flow rates in the range of liters per second. The results from one week of continuous CO_2_ flow observation from a wet mofette at the Starzach site is presented and correlated with the ambient meteorological dynamics. In the observed period, the gas flow rate of the examined mofette exhibits a dominant cycle of around four seconds that is linked to the gas rising upwards through a water column. We find the examined mofette to have a daily emission of 465 kg ±16 %. Furthermore, two events were observed that increased the flow rate abruptly by around 25 % within only a few minutes and a decaying period of 24 hours. These types of events were previously observed by others at the same site but dismissed as measurement errors. We discuss these events as a hydrogeological phenomenon similar to cold-water geyser eruptions. For meteorological events like the passages of high pressure fronts with steep changes in atmospheric pressure, we do not see a significant correlation between atmospheric parameters and the rate of gas exhalation in our one-week time frame, suggesting that on short timescales the atmospheric pumping effect plays a minor role for wet mofettes at the Starzach site.

## Introduction

Due to its increasing atmospheric concentration, carbon dioxide (CO_2_) currently has the largest bulk impact on total effective radiative forcing and is therefore the most relevant greenhouse gas (GHG) today (Forster et al., 2021), followed by methane (CH_4_) and nitrous oxide (N_2_O), which are more potent but less abundant greenhouse gases (Wallace & Hobbs, 2006). Under the globally adopted Paris Agreement (UNFCCC, 2015), countries are obliged to report annually on up-to-date GHG emission inventories to accepted standards (IPCC, 2006). The establishment of these inventories requires an array of methods, techniques and instruments to quantify gas fluxes over a variety of spatial scales. These range from in-situ point source estimation (Chiodini et al., 1998; Carapezza & Granieri, 2004; Lübben & Leven, 2022) to satellite remote sensing (Chevallier et al., 2005; Pan et al., 2021) and (global) inverse gas transport modelling for emission source back-tracing and budgeting (Pickett-Heaps et al., 2011; Gaubert et al., 2019). Ongoing GHG emissions and their consequences make it increasingly clear that negative emissions, e.g. in form of Carbon Capture and Sequestration (CCS), are needed to counter global warming (Gasser et al., 2015). Monitoring of CCS sites is important to ensure the security of CO_2_ storage  (Holloway, Pearce, Hards, Ohsumi, and Gale, 2007; Flohr et al., 2021), and surface monitoring techniques should be as mobile as possible (Jones et al., 2014). In general, uncertainty quantification is also desired and necessary for GHG emission estimations (Jonas et al., 2019).

In addition to anthropogenic causes, the earth mantle is another and permanent source of CO_2_ due to its degassing of the magma during crystallization (Lowenstern, 2001). The solubility of CO_2_ in the magmatic fluid decreases during crystallization (Dasgupta, 2013), resulting in magmatic CO_2_ exsolution which is then eventually capable of rising to the surface. CO_2_ may enter the lower atmosphere e.g., through active or dormant subaerial volcanos, fumaroles, mofettes, at mid-ocean ridges, geothermal systems and geysers (Glennon & Pfaff, 2005; Kerrick, 2001; Werner & Cardellini, 2006; Werner et al., 2019). Although these non-anthropogenic CO_2_ emissions are estimated to be two orders of magnitude smaller than anthropogenic emissions (Burton et al., 2013), they remain an integral baseline of the earth’s GHG budget. Past research has shown repeatedly that estimates for the total volcanic CO_2_ emissions vary greatly and better quantification is needed (Kerrick, 2001; Chiodini et al., 2004; Burton et al., 2013). Furthermore, such degassing can impact crop or forest growth (Farrar et al., 1995) and be hazardous to lifestock or humans (Beaubien et al., 2003). Temporal degassing anomalies around volcanos also show promising potential as precursors of volcanic eruptions (Inguaggiato, Vita, Cangemi, and Calderone, 2020; Pérez et al., 2022), and could improve the still insufficient early-warning systems (Winson et al., 2014). Therefore, the advancement of quantification methods for natural degassing from the solid earth remains an important task.

There exist several in-situ and remote sensing methods to quantify degassing from the solid earth, each suitable for one specific use case. While approaches to estimate gas *flux* (amount per area and time) vary, the vast majority of methods use spectrometry to quantify the gas  *concentration*.

Satellite data provides coarse global gas concentration data (Chevallier et al., 2005; Pan et al., 2021). One-dimensional column measurements of sulfur dioxide (SO_2_) on scales up to several kilometers are performed with remote sensing spectrometry that use the solar spectrum as a reference, such as correlation spectroscopy (COSPEC) (Williams-Jones et al., 2008) and its more compact iterations FlySPEC (Horton et al., 2006) and mini-DOAS (Galle et al., 2003; McGonigle et al., 2002), which give comparable results (Elias et al., 2006). Given further assumptions and boundary conditions such as the wind speed, these measurements can be translated into a gas flux or be used as proxy for other gases such as CO_2_ if not directly measured (Williams-Jones et al., 2008). However, the equipment for these techniques is rather expensive and requires careful operation and frequent calibration. Furthermore, a direct line of sight to sunlight is required, preventing its use during the night or in constrained locations. This also makes it less suitable for small, focused degassing point sources or weak diffuse degassing. There exist also similar laser or Fourier-Transform Infrared Spectroscopy (FTIR)-based approaches (Feitz et al., 2018) and local modelling techniques to merge and unify data from different sources (Feitz et al., 2022).

For diffuse degassing from soil or cropland, in-situ measurements are typically employed. A versatile technique suitable for homogeneous, flat terrain with a horizontal footprint up to hundreds of meters is the eddy-covariance method for directly measuring the turbulent vertical gas exchange (Mauder et al., 2021). While the eddy-covariance method can deliver high-frequency flux data (up to 20 Hz), it is unsuitable for complex terrain or very heterogeneous surface emissions (Baldocchi, 2003; Scholz et al., 2021). To a degree, the high-frequency data availability can be traded for lower cost by employing the flux-gradient approach, where the vertical gradient of slower gas concentration measurements is parameterised to yield an average flux, though losing precision. However, this method requires knowledge, calibration or approximation of the eddy diffusivity *K* and its dependence on atmospheric conditions (Zhao et al., 2019).

Another in-situ method for diffuse soil gas flux quantification is the dynamic concentration method (Gurrieri & Valenza, 1988; Camarda et al., 2019). Here, gas is pumped from the soil with increasing intensity until a constant gas concentration is sampled, signaling an equilibrium between pump flow and soil gas flux. While comparably simple to execute, this method is prone to overestimation and very dependant on soil permeability according to Carapezza and Granieri (2004). Instead, the accumulation chamber technique has proven to be a powerful alternative (Chiodini et al., 1998; Haro et al., 2019) by deriving a flux from the rate of concentration increase in a closed volume above the soil of interest.

However, the above-mentioned methods have been developed to investigate mainly diffuse degassing and so none of them is capable of directly quantifying advective gas fluxes of intense gas exhalations such as fumaroles or mofettes as the flow rates are either too high or the exhalations too focused. For strong advective degassing from vents, a robust method is to channel the exhaled gas and measure its velocity and concentration to determine the mass flow (Rogie et al., 2000; Lübben & Leven, 2022). However, to our knowledge, no such design has been published that focuses on continuous, unattended operation, high temporal resolution, low-cost components and adaptability to different magnitudes of degassing. In this study, we present a system with such potential. We assess the suitability of each individual component and demonstrate it by short-term application to a mofette at the Starzach site in Germany (Lübben & Leven, 2018). The degassing behaviour of the investigated mofette is discussed and a first, preliminary look is taken at the effects of meteorological parameters such as atmospheric pumping  (Nilson et al., 1991; Forde et al., 2019).

## Geological setting of the test site

**Fig. 1:**
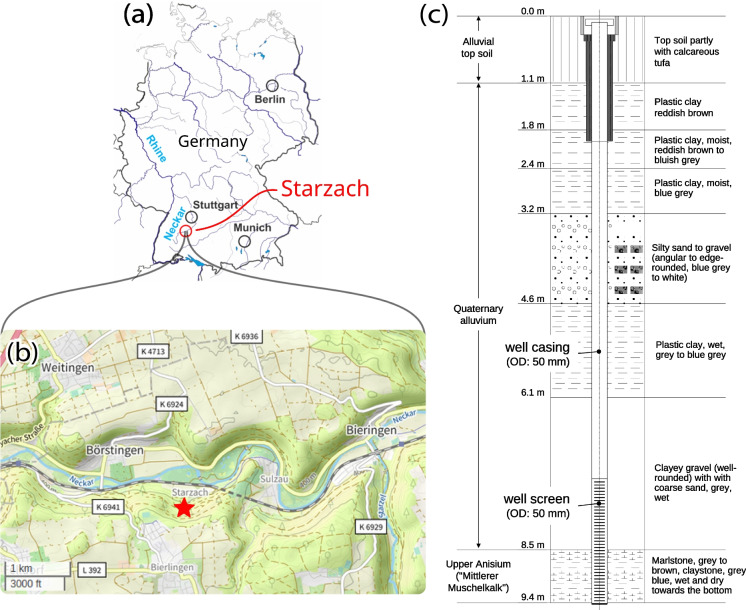
Overview of the Starzach site. (**a**) geographic location in Germany. (**b**) local map of the Neckar valley (OpenStreetMap contributors, 2023). (**c**) well log of a groundwater monitoring well at the Starzach site (Fig. 2a) with lithological description. The first ${\sim }6.4$ m of the well pipe are unscreened, while the lower ${\sim }3\,\text {m}$ are screened to access the groundwater

The Starzach site (Fig. 1) is located in Southwest Germany in the Upper Neckar valley, approximately 30 km southwest of Tübingen. In this region, the River Neckar cuts deep into the competent limestone of the Middle Triassic (“Muschelkalk”) forming a valley with relatively steep hillslopes formed by hillside depris covering the rock faces of the Middle Triassic. The site itself is located at the bottom of the Neckar valley, and is known for its natural CO_2_ degassing from mofettes and springs. In the region, CO_2_ was mined industrially over the last century until yields eventually declined, and stricter environmental regulations rendered the mining uneconomical. After a recovery period, degassing activity has increased again in the last decades, motivating current research activities in the area, for which Lübben and Leven (2018) introduced the Starzach site as a natural analog for leaking CCS sites. Their investigations show that the active gas exhalations are most likely linked to a fault zone following the major tectonic Swabian-Franconian direction, and that the emitted gas is most likely of non-volcanic magmatic origin consisting of a mixture of CO_2_ (${>}98\,\%$), nitrogen (${\sim }1$ %), oxygen (${\sim }0.2$ %) and smaller amounts of helium, argon and methane. A detailed description of the site and its geological setting is given in Lübben and Leven (2018).

**Fig. 2:**
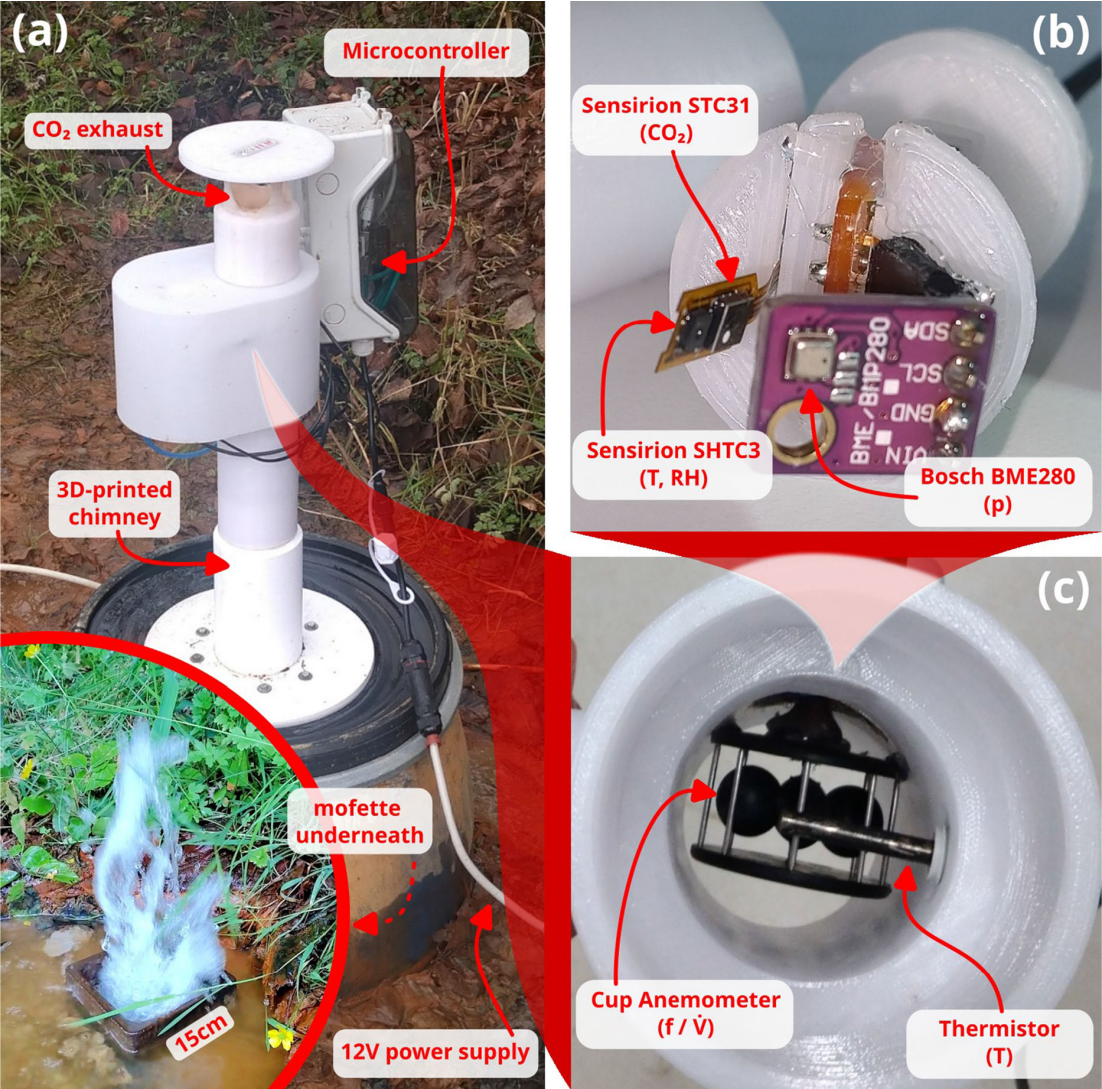
The chimney-based design to measure advective CO_2_ fluxes from mofettes. **(a)** Assembled hood deployed at the Starzach site over an erupting mofette as shown in the small inset and in Figure 2c of Büchau et al. (2022). Note: This is a different mofette than the one examined by Lübben and Leven (2022). **(b)** Gas sensor unit mounted laterally in the chimney consisting of Sensirion STC31 CO_2_ sensor, Sensirion SHTC3 temperature and humidity sensor and Bosch BME280 absolute atmospheric pressure sensor. **(c)** View from below through the chimney with the fitted cup anemometer and thermistor visible

A groundwater well was installed in May 2014 at a location without natural CO_2_ degassing for access to groundwater  (Figs. 1c and 2a). The 2”-well (DN50) targets the transition of the Quaternary aquifer to the Triassic bedrock unit (“Middle Muschelkalk”, Middle Triassic, Upper Anisium) and reaches a depth of 9.4 m, while the lowermost 3 m of the well are screened to access the groundwater. The undisturbed water level in the well after its completion was approx. 1.7 m below ground surface. At the time of installation, the well did not emit any noticeable amount of gas, but turned into a mofette approximately six months after, and the gas exhalation increased over the years through the well. Simultaneously, an adjacent smaller mofette in a distance of approx. 2 m disappeared over the years, and likewise the exhalation activity declined visibly at the larger mofette “R” (Lübben & Leven, 2018). This indicates a small-scale shift in the underground gas flow, a change contributing to the temporal and spatial heterogeneity of atmospheric CO_2_ concentration at the site. Lübben and Leven (2022) presented a custom funnel flow meter with which they determined flow rate magnitudes in the order of a few liters per second from specific mofettes such as mofette “R” at the site in 2015.

Recently, Büchau et al. (2022) deployed a wireless sensor network at the site to monitor atmospheric CO_2_ concentration and meteorological parameters and to provide infrastructure for further measurements. A strong diurnal cycle in atmospheric CO_2_ concentration was observed with typical, low baseline concentrations of range 400 ppm to 500 ppm during the day and strongly elevated concentrations up to 40 000 ppm during the night, caused by a lack of wind.

## Methods

### Chimney design

Chimney-based designs to measure advective gas fluxes from mofettes were already introduced by Rogie et al. (2000) and Lübben and Leven (2022). However, those setups are not suitable for prolonged continuous monitoring. Both applied a hot-wire anemometer to measure flow velocity and expensive infrared gas analysers for the gas concentration. Lübben and Leven (2022) found that the exact placement of their hot-wire anemometer inside the chimney had a strong impact on the estimated gas flux. Furthermore it was susceptible to measurement errors due to water deposition on the weakly heated element.

The design we present here addresses these problems: We focus on reduced cost, continuous operation, low power consumption and the ability to record data with high temporal resolution ($\Delta t < {1}$ s) to study the flow dynamics of the gas source.

With a chimney-based funnel design, given the volumetric gas flow rate $\dot{V}~\left[ \text {m}^{3}\,\text {s}^{-1}\right]$, the volumetric concentration of the gas of interest  $X_\textrm{gas}~\left[ \textrm{ratio}\right]$ (in this case for CO_2_:  $X_\mathrm {CO_2}~\left[ \textrm{ratio}\right]$), the temperature $T~\left[ \text {K}\right]$ and pressure $p~\left[ \text {Pa}\right]$ in the chimney, the mass flux $\dot{m}_{\mathrm {CO_{2}}}~\left[ \text {kg}\,\text {s}^{-1}\right]$ can be calculated with

1 $$\begin{aligned} \dot{m}_\mathrm {CO_2} = X_\mathrm {CO_2} \cdot \dot{V} \cdot \frac{ p \cdot M_\mathrm {CO_2}}{ R^*\cdot T } \end{aligned}$$

where $R^*\approx {8.314}\,\text {JK}^{-1}\,\text {mol}^{-1}$ denotes the universal gas constant and $M_\mathrm {CO_2} \approx {0.044}\,{\text {kg}\,\text {mol}^{-1}}$ the molar mass of $\text {CO}_{2}$.

To quantify the CO_2_ mass flux $\dot{m}_\mathrm {CO_2}$, the volumetric flow rate $\dot{V}$, the volumetric CO_2_ concentration $X_\textrm{CO2}$, gas pressure *p* and temperature *T* need to be measured. In the following we detail the respective sensors we employ and the calibration procedures we performed to validate those.

### Flow rate $\dot{V}$ measurement

Anemometry techniques to measure air flow velocity have evolved to a variety of choices for different applications and environments, from simpler working principles like pitot tubes, vane and cup anemometers, to sophisticated techniques such as hot-wire, ultrasonic or laser-Doppler anemometry (Foken, 2021; Camuffo, 2019). For our application of measuring the gas flow velocity of advective gas emissions, a small anemometer fitting into a tube with a diameter of a couple of centimeters is desirable. Anemometers that measure the flow velocity independently of the medium’s composition are especially favorable for the case of gas mixtures. In addition, robustness against water droplets, dew and elevated water vapour concentration in general is necessary to withstand the extremely humid conditions in the gas exhaled from a wet mofette. This rules out hot-wire anemometers as they are delicate devices mostly suitable for lab environments. Differential pressure sensors needed for Pitot tubes or other pressure-based flow rate measurement approaches are often designed for dry conditions only. Pitot tubes and vane anemometers must be calibrated or corrected for density (Foken, 2021). While ultrasonic and laser-Doppler anemometers are fundamentally independent of the medium by their physical design principles (Foken, 2021), commercially available devices are expensive and often large. A good balance between cost and medium-independence is the cup anemometer: In the simplified model of a two-cup anemometer, as it reaches a constant rotation frequency *f* in a stationary flow of velocity *v*, the opposing drag forces $F_{\text{ c }x}$ and $F_{\text{ c }v}$ acting on the convex and concave cup side, respectively, are at an equilibrium:

2 $$\begin{aligned} {\begin{matrix} F_{\text{ c }x} & = F_{\text{ c }v} \\ \frac{1}{2} \, A \, \rho \, c_{\text{ w },cx} \, \left( 2 \pi f r \!+\! v \right) ^2 & \!=\! \frac{1}{2} \, A \, \rho \, c_{\text{ w },cv} \, \left( 2 \pi f r \!-\! v \right) ^2 \end{matrix}} \end{aligned}$$

where the medium density $\rho$ and the cups’ cross-sectional area *A* cancel out. This leaves the rotation frequency *f* a sole function of the flow velocity *v* and the design parameters (the cup sides’ drag coefficents $c_{\text{ w },cx}$ and $c_{\text{ w },cv}$ and the cup centers’ distance *r* from the rotation axis). The intrinsic difference in drag between the shells, however, causes faster acceleration than deceleration and thus a hysteresis in rotation frequency in unsteady flows due to inertia, often referred to as overspeeding (Busch and Kristensen, 1976; Papadopoulos et al., 2001). Still, a cup anemometer can be a cost-effective way of measuring the gas flow rate inside a pipe independently of the gas composition as the influence of the overspeeding effect can be controlled for by comparison with reference measurements.

Small-sized cup anemometers are less common and mostly available as handheld devices which are unsuitable for automated continuous data logging. So we detached the protective cage containing the rotating cups from a commercially available handheld device. As is common for miniature cup anemometers, our model (Fig. 2c) has an axle with pointed ends sitting in metal sockets. This minimizes the amount of moving parts and friction contacts in comparison with e.g. a needle bearing, thus reducing the chance of failure under condensing conditions. We added an infrared light-emitting diode (LED) and a photodiode to act as light barrier for detecting the rotation frequency of the cups. The inverse of the pulse time divided by the amount of cups (four in this case) is then the cup anemometer’s rotation frequency. A microcontroller finds the pulse edges and records the time in between. As a consequence, the data rate for the cup anemometer’s rotation frequency is not constant as it depends on the rotation frequency itself.

Instead of parameterising the flow rate $\dot{V}$ as the product of cross-sectional area *A* and flow velocity *v* ($\dot{V} = A \cdot v$, cf. Lübben and Leven (2022); Rogie et al. (2000)), we calibrated our system as a whole to translate the rotational frequency *f* of the cup anemometer directly to the flow rate $\dot{V}$. This avoids that the effective cross-sectional area might be unknown due to the geometry of the chimney and flow obstructions such as the anemometer itself. Furthermore, friction causes the flow velocity to diminish near the walls of the chimney, resulting in a lateral velocity profile instead of a constant value across the cross-section, which is an implicit assumption for the parametrisation $\dot{V} = A \cdot v$. This effect is increased with smaller Reynolds numbers as the velocity peak in the center of the chimney becomes more prominent (Etling, 2008; Štigler, 2012). The Reynolds number for a setup like ours (55 mm inner chimney diameter, CO_2_, 1 m s$^{-1}$ velocity) ranges from 4500 to 10 000, taking into account variations in temperature, pressure (Schäfer et al., 2015; Foken, 2021), flow velocity and dimensional uncertainties. Considering that the flow through the chimney is obstructed by sensors and a protective water shield at the inlet and outlet (Fig. 2), it is reasonable to assume the chimney flow will not be laminar but weakly turbulent, unifying the velocity throughout the cross-section.

We carried out two experiments to ensure our flow rate measurement is valid. First, to determine the relationship between *f* and $\dot{V}$ we connected our chimney to an LTG 227VM-05 volumetric flow sensor that is part of our research facility’s building ventilation system and recorded the cup anemometer’s rotational frequency *f* while varying the flow rate by gradually closing the shutt-off valve of the ventilation. Second, in the field we repeatedly took the time it takes to fill up plastic bags of known volume with gas from a mofette and compared this to the estimate derived from the lab results. These results are discussed in Section 4.1.

### CO_2_ measurement $X_\mathrm {CO_2}$

A CO_2_ sensor for measuring advective CO_2_ fluxes from mofettes needs to fulfil several criteria: First, it needs to be able to measure high CO_2_ concentrations close to 100 % (Lübben & Leven, 2018; Büchau et al., 2022; Lübben & Leven, 2022). It also has to be small enough for fitting into a chimney next to the other sensors. A reasonably high measuring frequency ($\ge {1}{\text {Hz}}$) is necessary if dynamics of flow rate and gas concentration are to be analysed. Finally, extremely humid environments should neither harm the sensor nor influence the measurement too strongly. This combination of requirements is rather unusual and the market offer of the gas sensor industry is quite limited in this regard. Many embedded non-dispersive infrared (NDIR) CO_2_ sensors suffer from the cross-sensitivity on water vapour, have slow response times and can only measure low CO_2_ levels (Müller et al., 2020; Büchau et al., 2022). Initial tests with a GSS ExplorIR-M NDIR CO_2_ sensor which can measure up to 100 % CO_2_ were unsuccessful under very humid conditions.

Another approach to measure gas concentrations is using a proxy quantity that is strongly influenced by the gas mixture (e.g. sonic speed or heat conductivity) and deducing a concentration given assumptions and further information about the gas composition. The Sensirion STC31 CO_2_ sensor is such a model which derives a CO_2_ concentration from the heat conductivity. Compared to other embedded CO_2_ sensors such as those evaluated in Büchau et al. (2022), the STC31 sensor is an order of magnitude smaller with a size of only ${3}\,{\text {mm}} \times {3.5}\,{\text {mm}} \times {1}\,{\text {mm}}$ (Fig. 2b). Furthermore, the STC31 sensor covers the entire CO_2_ concentration range from 0 % to 100 % – a capability most comparable NDIR-based CO_2_ sensors lack (Büchau et al., 2022).

The STC31 sensor needs to have the temperature, pressure and relative humidity communicated to it before it performs a measurement, then internally calculates and reports a CO_2_ concentration. We employ an evaluation kit where a Sensirion SHTC3 temperature and humidity sensor is mounted directly next to the STC31 CO_2_ sensor (Fig. 2b). Readings of the former sensor are communicated to the STC31 CO_2_ sensor. The pressure measurement is performed by a Bosch BME280 environmental sensor, a common miniature low-cost absolute atmospheric pressure sensor with a rated absolute accuracy of around  ${\pm 1.5}\,{\text {hPa}}$ (Fig. 2b). During operation we disable the STC31 sensor’s automatic self-calibration to prevent it from wrongly interpreting the high CO_2_ concentration as an implicit baseline.

To assess the STC31 sensor’s suitability we exposed it to various combinations of temperature, relative humidity and CO_2_ concentration inside an EdgeTech RH CAL relative humidity calibration chamber together with the intake of a LI-COR 840A closed-path infrared gas analyser. An automated gas injection system flooded the calibration chamber periodically with CO_2_ about every 30minutes after each successful transition to the next temperature/relative humidity level. The LI-COR sensor’s calibration range only reaches up to 20 000 ppm (2 vol%). However, its maximum data output limit is as high as 200 000 ppm (20 vol%). So for comparison with the LI-COR sensor, we capped the CO_2_ concentration during flooding of the calibration chamber at this level to reduce the idle time where no overlapping data within its calibration range is available. LI-COR measurements beyond 2 vol% are expected to have a larger error, but are nevertheless included here for reference.

To account for high CO_2_ concentrations, the same temperature and relative humidity profile was repeated but with periodic CO_2_ injections without an upper concentration limit. Furthermore, a separate setup with the STC31 sensor in the gas volume at the top of a bottle with carbonated water was performed to simulate saturated humidity and CO_2_ conditions similar to the situation in the field. The results are discussed in Section 4.2.

### Temperature *T* and humidity *RH* measurement

Two temperature measurements are installed in the chimney device; one measurement close to the CO_2_ sensor laterally in the chimney (small SHTC3 temperature and humidity sensor as described above, Fig. 2b) and one measurement right in the center of the chimney to record the actual temperature of the emitted gas without outside influence. For the latter measurement we use a positive temperature coefficient (PTC) thermistor in a metal housing for durability. Both sensors were calibrated in our RH CAL calibration chamber. The results are discussed in Section 4.3.

### Field measurements

Having calibrated the individual sensors, field tests were carried out at the Starzach site (Section 2). The mofette that developed from a groundwater monitoring well (Fig. 1c) was chosen for the measurements described here (Fig. 2a, same mofette as Figure 2c in Büchau et al. 2022).

A wireless sensor network is presently deployed at the Starzach site (Büchau et al., 2022). Sensor stations send data via a Wireless Local Area Network (WLAN) established by a central single-board computer with cellular internet access. Data is stored on µSD-cards on each sensor station as well as the central station and an off-site server where data is relayed to. Currently, all devices are powered from one 12 V lead-acid battery charged by a series of solar panels and a methanol fuel cell for backup, but every station could be powered independently to increase mobility. The chimney device itself has an average power consumption of around 0.5 W and was integrated into this network as a sensor station for one week of continuous operation. Data of a Gill MaxiMet GMX541 compact weather station located at the central station is available as meteorological reference. The obtained measurements are discussed in Section 4.4.

## Results and discussion

As a measure of similarity between two quantities *x* and *y* we employ the Mean Absolute Error (MAE):

3 $$\begin{aligned} \text {MAE}\left( x,y\right) = \text {mean} \left( \left| {\text {x} - \text {y}} \right| \right) \end{aligned}$$

For conservative sensitivity analysis, the maximum absolute error $\Delta y_\text {max}$ and maximum relative error $\Delta y_\text {max,rel}$ $\left[ {\%}\right]$ of a quantity *y* derived from input quantities $x_1, \dots , x_\text {n}$ can be calculated via

4 $$\begin{aligned} {\begin{matrix} \Delta y_\text {max}(x_1, \dots , x_\text {n}) & = \sum _{i=1}^n \left| \frac{\partial y}{\partial x_\text {i}} \right| \cdot \Delta x_{\text {i}_\text {max}} \\[1em] \Delta y_\text {max,rel} & = \frac{\Delta y_\text {max}}{\overline{y}} \end{matrix}} \end{aligned}$$

where $\Delta x_{\text {i}_\text {max}}$ is the maximum expected absolute error of quantity $x_\text {i}$ and $\overline{y}$ the mean of *y*.

### Flow rate $\dot{V}$ calibration

**Fig. 3:**
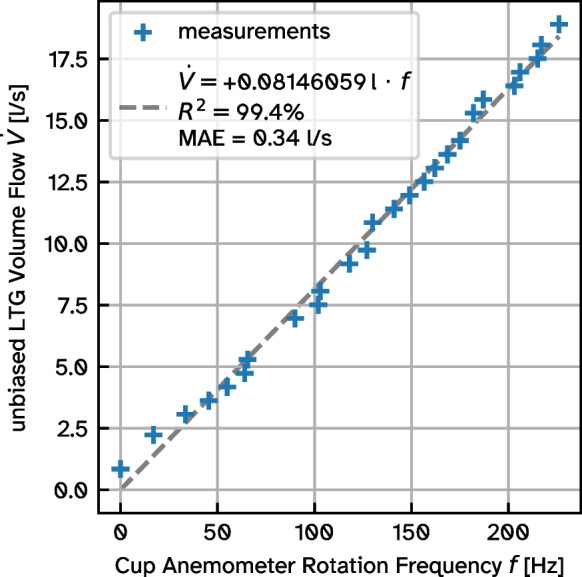
Calibration of cup anemometer rotational frequency inside chimney against flow rate of LTG 227VM-05 volumetric flow sensor

Comparing the rotational frequency of the cup anemometer installed in the chimney (Fig. 2c) to the volumetric flow rate obtained from an LTG 227VM-05 volumetric flow sensor under laboratory conditions, we find a linear relationship (coefficient of determination $R^2 = 99.4\,\%$) with an average error of 0.34 Ls$^{-1}$ (Fig. 3). As expected of cup anemometers due to the initial friction in the mechanical bearing (Alfonso-Corcuera et al., 2021), flow rates below 3 $\text {Ls}^{-1}$ are slightly underestimated in our setup.

**Table 1:** Bag calibration data visualised in Fig. 4

Nr	Bag volume ${V~[\text {L}]}$	duration ${\Delta t~[\text {s}]}$	Flow rate ${\dot{V} [\text {Ls}^{-1}]}$
1	$50 \pm 20$	$27.6 \pm 1.0$	$1.4 \pm 0.8$
2	$100 \pm 30$	$26.7 \pm 1.0$	$4.1 \pm 1.3$
3	$240 \pm 50$	$78.3 \pm 1.0$	$3.1 \pm 0.7$
4	$240 \pm 50$	$81.8 \pm 1.0$	$2.9 \pm 0.6$
5	$240 \pm 50$	$75.0 \pm 1.0$	$3.2 \pm 0.7$
6	$240 \pm 50$	$75.1 \pm 1.0$	$3.2 \pm 0.7$
7	$60 \pm 20$	$22.2 \pm 1.0$	$2.7 \pm 1.0$

The uncertainties of bag volume and duration were estimated very conservatively from on-site dimensional and timing measurements and video footage of the experiments using Eq. 4, then translated into the flow rate uncertainty by applying Eqs. 4 to 5

With this relationship determined, we took the device to the field and installed it on a mofette (Fig. 2a). We removed the top chimney roof segment and repeatedly attached plastic bags with nominal volumes of 60 L, 120 L and 240 L to the exhaust of the chimney to fill them up with gas exiting from the mofette. The measured time $\Delta t$ it takes to fill up a bag of volume *V* was then used to calculate the average flow rate during the filling time period:

5 $$\begin{aligned} \dot{V} = \frac{V}{\Delta t} \end{aligned}$$

When inflated, the plastic bags had non-trivial shapes, so we estimated their volume very conservatively from dimensional measurements assuming a cylindrical shape as approximation. Applying Eq. 4 to Eq. 5 then also yields a propagated error estimation for the average flow rate. Data of the individual bag fills is listed in Table 1.

**Fig. 4:**
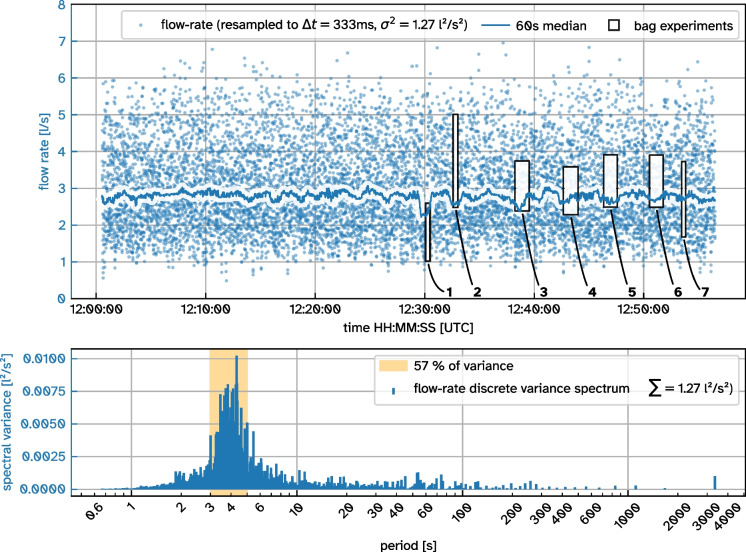
Time series of measured volumetric flow rate from the examined mofette (Fig. 2a) with a temporal resolution resampled to 3 Hz. Each outlined box indicates a bag fill detailed in Table 1. *Bottom*: Variance spectrum of the volumetric flow rate time series

During the bag fills we recorded flow rate data deduced from the cup anemometer’s rotational frequency at an average data rate of 3 Hz. This time series together with the flow rate estimation from the bag fills is plotted in Fig. 4. The observed flow rate varies between $1\,\text {Ls}^{-1}$ and $6\,\text {Ls}^{-1}$ with an average slightly below $3\,\text {Ls}^{-1}$.

When bags are attached to the chimney, the flow rate initially plummets and is then slowly restored during inflation. The drop in flow rate is especially prominent for the smaller bags 1 and 2. On initial contact between bag and chimney, the introduced orifice at the interface is limiting the flow. Furthermore, during inflation the bag foil needs to straighten from its wrinkled state, providing resistance for incoming gas. Both effects decrease in intensity the more the bag is inflated, allowing the flow rate to recover.

Due to the shorter filling times and uneven shapes of the smaller bags 1, 2 and 7, their flow rate uncertainties are the largest. Still, the flow rate deduced from the cup anemometer generally lies within the flow rate range estimated from the respective bag fill. This indicates that our lab calibration is correct and also applicable under field conditions.

A dominant cycle is present in the flow rate signal with a period of 4 seconds, responsible for more than half (57 %) of the total signal variance (Fig. 4, bottom). This 4 s-cycle corresponds to the observable bubbling that is characteristic for wet mofettes at the site and is visible in Fig. 2a and in c of Büchau et al. (2022). Our understanding of this 4 s-cycle is that it is caused by a periodically shifting pressure equilibrium within the well pipe shown in Fig. 1c. The gas ascends up to the point where the pipe perforation ends in 6.4 m depth. At this point, the water column maintains a hydrostatic pressure of ${\sim }63$ kPa when the well pipe is filled to the top. As more gas accumulates from below, this pressure is eventually overcome so that an eruption happens, releasing the built-up gas pressure. Measurements with a closed chimney exhaust showed maximum differences to atmospheric pressure of ${\sim }100$ kPa (1bar), which supports this explanation. Surrounding ground water constantly enters the well pipe through the perforation, refilling the water column. This cycle apparently repeats with a period of 4 s.

**Fig. 5:**
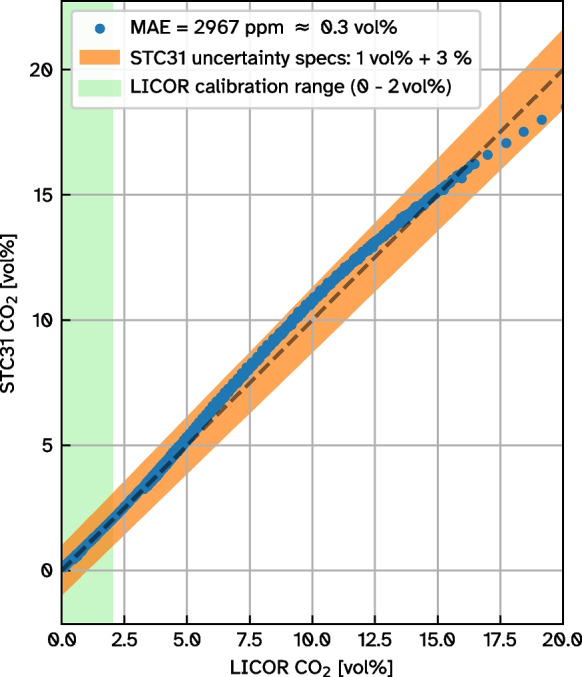
Comparison of LI-COR840A closed-path infrared gas analyser and STC31 heat conductivity CO_2_ sensor measurements in an EdgeTech RH CAL calibration chamber for various combinations of relative humidity and temperature

### CO_2_ Measurement $X_\mathrm {CO_2}$ Verification

In the calibration chamber setup detailed in Section 3.3, the temperature ranged from ${11}\, ^{\circ }\text {C}$ to ${40}\, ^{\circ }\text {C}$. Due to the periodically injected dry CO_2_ gas, the calibration chamber struggled generating very humid conditions, resulting in a range of generated relative humidity from 6 % to 74 %. Under these conditions, both CO_2_ sensors (STC31 and LI-COR 840A) agree very well over the entire LI-COR output range up to 20 vol% with a mean absolute error of 0.3 vol%, even beyond the LI-COR sensor’s calibrated range where the relationship becomes non-linear (Fig. 5). The non-linear relationship above 2 vol% can not be explained with a mismatch in response times of the two sensors - filtering either sensor with an optimized exponentially-weighted moving average (EMWA) did not result in any significant linearization. Still, the deviation between both sensors lies within the STC31 sensor’s specifications and is only weakly correlated with chamber temperature (21 %) and relative humidity (-18 %). These two sensors have fundamentally different measuring principles (LI-COR: infrared absorption vs. STC31: heat conductivity) and it is unlikely that both are biased identically. As a consequence, the good aggreement between the two indicates that the LI-COR sensor’s measurements can still be relied upon beyond its calibrated range, though with a larger margin of error.

During the 23 periodic full CO_2_ floodings of the calibration chamber the CO_2_ concentration peaks measured by the STC31 sensor had an average magnitude of 97.6 vol% and a maximum of 99 vol%. A slightly lower result than full CO_2_ saturation is expected as the calibration chamber constantly feeds outside air into the volume for purposes of mixing the humid air, thus diluting the introduced CO_2_. This result proves that the STC31 sensor can reliably measure high CO_2_ levels under dry conditions.

**Fig. 6:**
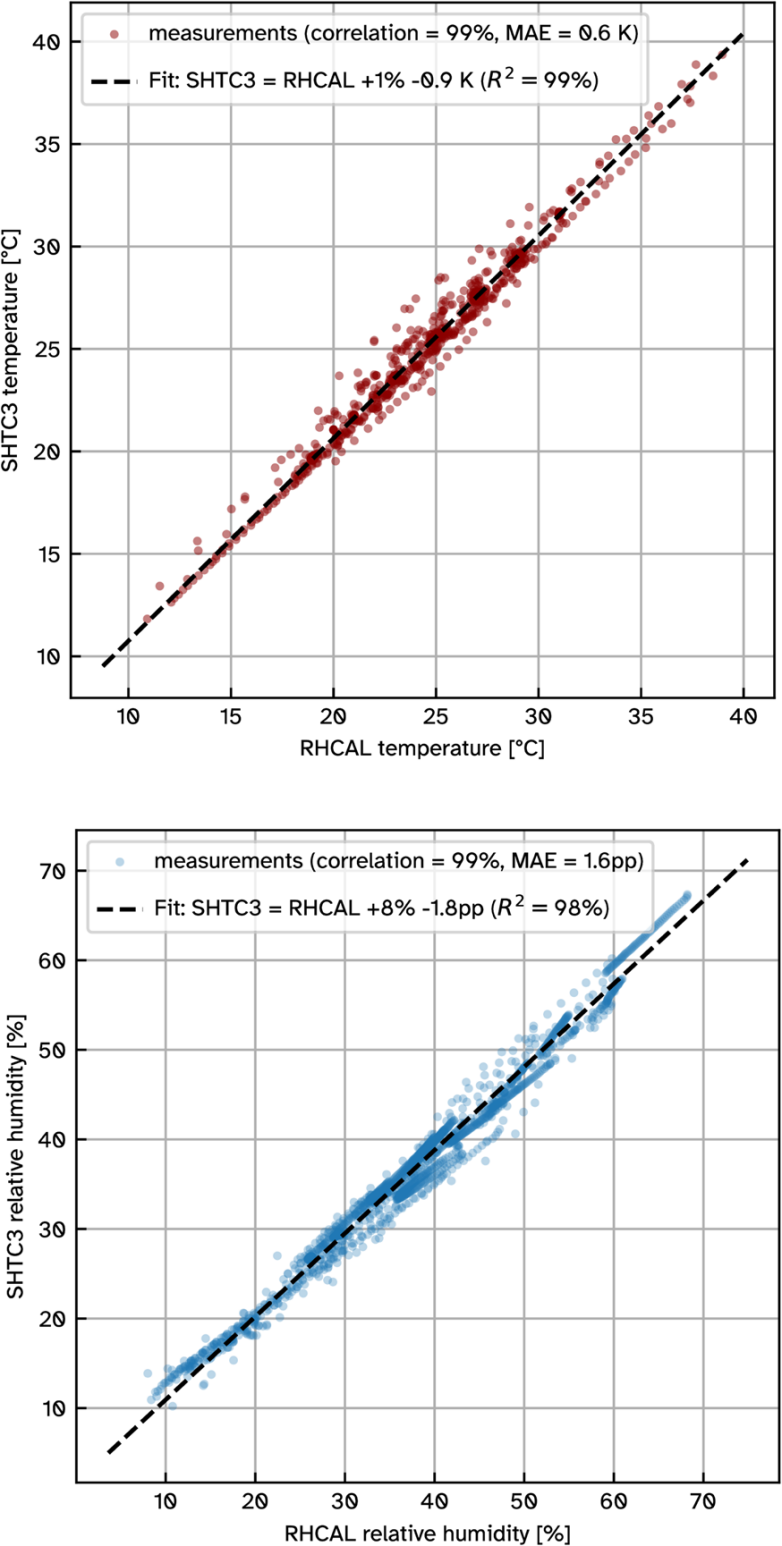
Verification measurements of SHTC3 sensor inside RH CAL calibration chamber for temperature (top) and humidity (bottom). The data was obtained in the same setup as in Fig. 5

A matching measurement of 99.4 vol% was obtained in the gaseous volume of the carbonated water bottle. We allowed the gas phase to reach an equilibrium for three hours, approaching full saturation of the mixture of water vapour and CO_2_; similar conditions to what we expect to find in the field. From SHTC3 and BME280 measurements ($T=20\, ^{\circ }\text {C}$, $RH = 83\,\%$, $p=978\,\text {hPa}$) it can be estimated that water vapour should take up ${\sim }2$ vol% of the mixture, leaving ${\sim }98$ vol% for CO_2_. For simplicity of this estimation, we ignore the quite complex effects of dissolved CO_2_ on saturation water vapour pressure (Privat & Jaubert, 2014) here. The obtained CO_2_ concentration of 99.4 vol% still lies within the STC31 sensor’s uncertainty of ${\pm }$1 vol% ±3 %. Thus, in contrast to infrared CO_2_ sensors which can have a strong cross-sensitivity on water vapour (Büchau et al., 2022), the STC31 sensor is also suitable for humid conditions.

### Temperature *T* and humidity *RH* calibration

During the same calibration experiment as described above, the SHTC3 temperature and humidity sensor (Fig. 2b) was present to feed its data to the STC31 CO_2_ sensor. Comparing its data to the calibration chamber measurements (Fig. 6), an average accuracy of 0.6 K for temperature and 1.6 pp (percent points) for relative humidity is asserted across the entire experiment time series including the CO_2_ floodings.

**Fig. 7:**
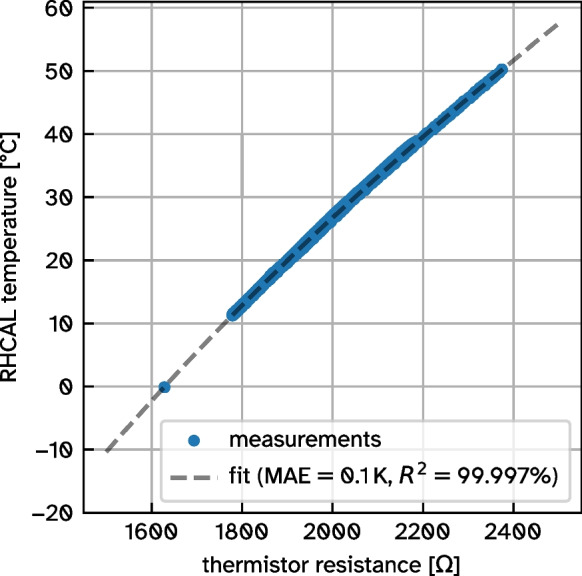
Thermistor calibration in reference to RH CAL calibration chamber with a polynomial fit of 3rd degree

In another independent setup, the thermistor (Fig. 2c) was calibrated in the calibration chamber. In addition to a temperature profile from the calibration chamber, one data point in ice water was added to increase the reference range. A polynomial fit of third degree describes the thermistor’s temperature dependency to an accuracy of 0.1 K (Fig. 7).

### Field measurements discussion

**Fig. 8:**
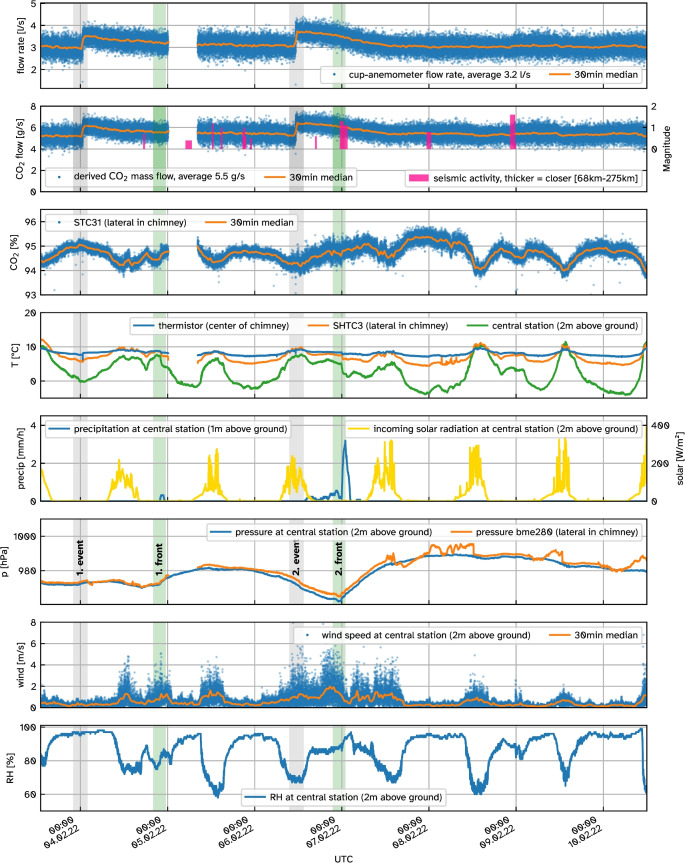
One week of continuous measurements of the chimney device mounted on a mofette (Fig. 2a) at the Starzach site at 10 s resolution. Meteorological data is provided by a Gill MaxiMet GMX541 compact weather station (“central station” in Büchau et al.  2022). Two front passages are marked as green vertical lines. Gray vertical lines indicate the times of the two flow rate events. In the first hours of the 05.02.2022 there was a data gap due to intermittent transmission problems. Seismic activity data was obtained from https://erdbeben.led-bw.de (Landeserdbebendienst, Landesamt für Geologie, Rohstoffe und Bergbau, Regierungspräsidium Freiburg, Baden-Württemberg, Germany) on 29.09.2023

An under-sampling analysis in the post-processing of the flow rate validation discussed in Section 4.1 showed that a 10 s sampling interval for the cup anemometer frequency measurement introduces an error of just ±1 % for the average flow rate compared to a sampling rate of 3 Hz. To keep network traffic low, we thus chose a data interval of 10 s for the continuous measurements. One week of data was recorded with the device mounted on the mofette shown in Fig. 2a. This data together with meteorological measurements from a Gill MaxiMet GMX541 compact weather station is shown in Fig. 8. Except for an 8 h data gap due to intermittent transmission problems in the night of the 05.02.2022, the instrument delivered data continuously.

#### Meteorological situation

The observation period took place in the late winter of 2022, from February 3^rd^ to 10^th^. Temperatures at 2 m height above ground regularly dropped below ${0}\, ^{\circ }\text {C}$ during night time and reached up to ${11}\, ^{\circ }\text {C}$ during the day. As the site is being situated at a northern slope of the river valley, incoming solar radiation is further reduced in the morning and evening (Büchau et al., 2022). Consequently, relative humidity was constantly elevated with a minimum of 60 %. Two cold air front passages with precipitation events were observed within the monitoring period, a weaker first front right before midnight between 04. and 05.02.2022 and a very distinct second front at midnight between 06. and 07.02.2022. Both fronts caused a significant temperature drop (${\sim }3$ K within 30 min), an intermittent increase in wind speed and a sudden increase in atmospheric pressure. The air mass trailing the second front raised the atmospheric pressure by nearly 30 hPa over the next day.

#### Measurement artifacts

There is a clear and opposite relationship between STC31 CO_2_ readings and all temperature measurements. The strongest correlation is -86 % with the thermistor temperature. Such a significant temperature dependence was not observed under laboratory conditions (Section 4.2, Fig. 5). An explanation for a *positive* correlation could have been that each eruption brings a new volume of CO_2_-rich gas which is also warmer than the atmosphere surrounding the chimney. The observed behaviour, by contrast, rather indicates an inadequacy of the thermal model implemented in the STC31 CO_2_ sensor for the gas mixture emitted by the mofette. The STC31 sensor must be configured to assume the remaining non-CO_2_ gas as either nitrogen (N_2_) or air (our setting). Other than CO_2_, the gas mixture emitted from the Starzach mofettes consists of nitrogen (${\sim }1$ %), oxygen (${\sim }0.2$ %) and smaller amounts of helium, argon and methane (Lübben & Leven, 2018). Furthermore, ${\sim }1$ % of water vapour is reasonable to assume with full saturation at ${10}^{\circ }\text {C}$, similar to our estimation for the carbonated water bottle experiment in Section 4.2. In total, these remaining gases sum up to ${\sim }2$ vol%, leaving ${\sim }98$ vol% for CO_2_. Even with the fluctuations introduced by the apparent temperature dependency, the CO_2_ readings remain within the sensor’s rated accuracy of 1 vol% ±3 %.

Readings of the BME280 atmospheric pressure sensor mounted laterally in the chimney (Fig. 2b) exhibit some artifacts starting at noon on 07.02.2022. We assume these to be caused by condensation on the sensor as it is not rated for extremely humid conditions. Other models such as the BMP384 or BMP585 could be promising alternatives with a protective layer of gel.

#### CO_2_ exhaust

Over the course of the observation period, our instrument measured an average baseline CO_2_ exhaust from the single mofette of $5.4\,\text {gs}^{-1}$ which extrapolates to $465\,\text {kg\,d}^{-1}$ (excluding the anomalies discussed below). Applying Eq. 4 to Eq. 1 yields that the maximum relative error of the mass flux $\Delta \dot{m}_\textrm{max,rel}$ can be estimated as the sum of relative errors of its independent variables:

6 $$\begin{aligned} \Delta \dot{m}_\textrm{max,rel}= &  \Delta T_\textrm{max,rel} + \Delta \dot{V}_\textrm{max,rel}\nonumber \\ &  + \Delta X_\mathrm {CO_2,max,rel} + \Delta p_\textrm{max,rel} \end{aligned}$$

Inserting values determined above, this maximum relative error sums up to $\Delta \dot{m}_\textrm{max,rel} \approx {\pm 16}\,{\%}$, to which our mass flow estimates are accurate with high confidence. At their examined mofette (the visually most prominent one at that time in 2015), Lübben & Leven (2022) determined average mass flow rates of around ${75}\,\text {kg\,d}^{-1}$, which is significantly smaller. Still, our notably larger estimate for the visually most striking mofette today could signify a general increase in degassing activity at the site – a trend that has been going on since industrial mining of the gas has stopped (Lübben & Leven, 2018).

#### Flow rate $\dot{V}$ anomalies

As highlighted in Fig. 8, two events were observed where the flow rate rapidly increased by ${\sim }25$ % within a few minutes and then gradually declined over ${\sim }24$ h to settle back to baseline. The first event happened around midnight between 04. and 05.02.2022 and a second one 60 h later at noon on the 06.02.2022. Excluding these events from averaging results in a 3 % underestimation of exhaled mass, motivating a continuous monitoring solution for wet mofettes with comparable dynamics. Similar anomalies were observed by Lübben & Leven (2022) at a different mofette “R” at the site (Lübben & Leven, 2018), but dismissed as measurement error, as the event was only monitored once in their time-series. The reproducibility of these measurements with a completely different system as ours suggests there is an underlying process causing these events. Timing and magnitude of seismic activity in the wider region appear to be largely unrelated to the occurrence of flow anomalies during the period observed. Though no record of groundwater levels is present for the site, the flow anomalies can be assumed to be unrelated to groundwater levels of the Quaternary aquifer, as they are mainly controlled by the water level changes in the adjacent River Neckar, and there are no other disturbances of the aquifer in the closer vicinity, such as water supply wells. Besides the leap in flow rate, several other anomalies were measured during such an event: Most prominently, both events coincided with a very short (${<}1$ min) but significant temperature drop of nearly 2 K measured by the thermistor mounted in the center of the chimney (Fig. 2c). Furthermore, right at the beginning of each event, one single measurement of a greatly reduced flow rate was recorded. A very brief and dramatic reduction in CO_2_ concentration to around 50 % (not visible in Fig. 8) is also noticeable at this time. A drop this large is unlikely to be a consequence of the sensor’s temperature dependence discussed above.

These observations suggest that the advective CO_2_ degassing at the Starzach site obeys cold-water geyser mechanics (Han et al., 2013), albeit less effectively. Only a few cold-water geysers are known globally, with the world’s most prominent CO_2_-driven cold-water geyser being located in Andernach, Germany (Glennon & Pfaff, 2005). The periodic eruptions of a cold water geyser originate from the saturation of a water-filled cavity, which is constantly being supplied with gas from below. Oversaturation of the dissolved gas eventually leads to exsolution and uprising of gas bubbles. This reduces the pressure exerted by the overlying water column and initiates a positive feedback as the reduced pressure favours even more exsolution, resulting in an eruption (Glennon & Pfaff, 2005; Han et al., 2013). Eruption intervals and durations of known cold-water geysers vary between minutes and hours (Glennon & Pfaff, 2005). Jung et al. (2015) found eruption intervals and durations to be roughly proportional for the Crystal Geyser (Utah, USA) while the factor changes over time. The flow rate anomalies we observed would accordingly correspond to eruptions with an interval of several days and a duration of one day. Han et al. (2013) found steep temperature drops during eruptions of the Crystal Geyser and explain those with Joule-Thomson cooling and endothermic CO_2_ exsolution. However, the temperature drops we saw here are intermittent. This, together with the brief dips in flow rate, suggest a different cause. The cup anemometer we utilize for flow measurement is inherently independent of flow direction (Section 3.2). However, both a complete temporary flow stop as well as a flow change to the opposite direction will cause its rotation to decrease – albeit briefly. The latter seems to be the case here: As the mofette changes from exhaling to inhaling, cold surrounding air is transported inside the chimney to the thermistor, explaining both its measured temperature drop and the decrease in CO_2_ concentration. Apparently, this flow direction change happens over a short time period of 5 s to 15 s, as for both events only exactly one of the 10 s-spaced measurements captures it.

Lübben & Leven (2018) present a conceptual geologic model in which the claystone of the *Röt Formation* at the top of the *Upper Buntsandstein* in approx. 50 m depth acts as an impermeable barrier and therefore as a capstone for the uprising gas. Below, CO_2_ ascends through the water-saturated sandstones of the *Middle and Lower Buntsandstein*, which presents a potential reservoir for gas accumulation. Tectonic faults through the *Röt Formation* and the *Lower Muschelkalk* act as relatively undisturbed pathways to the surface and eventually to our examined well (Fig. 1c). We assume the oversaturation of water with CO_2_ happens initially below the *Röt Formation* in the reservoir. However, longer time series and further research is needed to further quantify this process.

#### Influence of meteorological parameters

The data obtained during our observation week does not suggest any significant connection between meteorological parameters and the degassing behaviour. The two flow rate anomalies described above do not coicide with any change in temperature, atmospheric pressure, precipitation or other atmospheric variables we recorded. In general, pressure inside the chimney closely follows atmospheric pressure measured at the central station. This is expected for a chimney diameter this large as no significant pressure is built up. Atmospheric pressure is known to influence diffuse degassing via the “atmospheric pumping” effect (Nilson et al., 1991; Forde et al., 2019) or change geyser eruption activity (Rinehart, 1972). Nevertheless, neither of the two cold air front passages resulted in an immediately noticeable variation in exhaled gas amount. However, when comparing the settling times it took to return to baseline flow after an event, a slightly faster decline can be observed after the second event, immediately after the second front has passed. This is a plausible connection given that the final 30 hPa pressure increase the second front introduced should correspond to an additional virtual ${\sim }30$ cm water column the ascending gas needs to overcome for an eruption, effectively reducing the flow rate. But the short time series we recorded here is insufficient to quantify this. Longer measurement periods spanning multiple seasons are needed to further investigate this effect.

## Conclusion and outlook

Chimney-based designs are well suited to continuously monitor degassing from vents. We introduced a low-cost, portable chimney device for continuously monitoring advective degassing from a mofette. An examined mofette was found to exhale 465 kg  ±16 % of CO_2_ per day, a result that is in line with previous measurements at the site (Lübben & Leven, 2022). During a short observation period of one week, meteorological parameters such as atmospheric pressure were found to have no immediate effect on the degassing behaviour, even during significant events as cold-front passages with steep atmospheric pressure changes.

Contrary to existing designs, our volumetric flow rate measurement is density-independent and can thus be used for a variety of other gases and gas mixtures. Being developed for continuous operation, this instrument is suitable to monitor long-term changes such as the observed shift of degassing intensity from one mofette to another or geyser-like eruptions happening on different time scales. Finding a correlation to earthquake activity is another reasonable application (Rinehart, 1972; Han et al., 2013; Woith et al., 2023). This could be especially interesting for the Starzach site where small-magnitude earthquakes happen occasionally in the region.

For degassing of greatly different output magnitudes, the 3D-printed chimney can be easily reprinted with an appropriate diameter, followed by a recalibration of the flow rate according to the procedure we described here. The adapter from chimney to vent (a 50 cm-diameter cut-open plastic barrel in Fig. 2a) can also be chosen freely, for example by 3D-printing a custom cup or even employing flexible material such as used by Rogie et al. (2000).

An improvement of the temporal resolution could be achieved by introducing a pinhole disk in the chimney and deriving the flow rate from the difference in pressure before and after the constriction (Bentley, 2005). The density-dependence of this approach needs to be accounted for, though. Another possibility is to integrate a custom 1D-ultrasonic anemometer into the chimney, which can measure the flow velocity independently of the gas by design. In general, utilization of waterproof pressure sensors such as the Bosch BMP384 or BMP585 is preferrable. Furthermore, local on-device storage of the data on a memory card can be implemented if offline operation is desired. For future flow rate calibrations using a similar bag-filling technique as demonstrated in this paper, we suggest using foil-balloons of a more quantifiable geometric shape (e.g. a sphere) with a large diameter (e.g. ${>}50$ cm) to decrease the volumetric uncertainty.

## Data Availability

Field data is available from PANGAEA Büchau et al. (2023).

## Code availability

Code is available at https://gitlab.com/tue-umphy/co2mofetten.

## Declarations

All authors have read, understood, and have complied as applicable with the statement on “Ethical responsibilities of Authors” as found in the Instructions for Authors.

## References

[CR1] Alfonso-Corcuera, D., Pindado, S., Ogueta-Gutiérrez, M., & Sanz-Andrés, A. (2021). Bearing friction effect on cup anemometer performance modelling. *Journal of Physics: Conference Series* (vol. 2090, p.012101). 10.1088/1742-6596/2090/1/012101

[CR2] Baldocchi, D. (2003). Assessing the eddy covariance technique for evaluating carbon dioxide exchange rates of ecosystems: past, present and future. *Global Change Biology,**9*(4), 479–492. 10.1046/j.1365-2486.2003.00629.x 10.1046/j.1365-2486.2003.00629.x

[CR3] Beaubien, S., Ciotoli, G., & Lombardi, S. (2003). Carbon dioxide and radon gas hazard in the Alban Hills area (central Italy). *Journal of Volcanology and Geothermal Research,**123*(1–2), 63–80. 10.1016/S0377-0273(03) 00028-3. Retrieved from https://www.sciencedirect.com/science/article/pii/S0377027303000283

[CR4] Bentley, J. P. (2005). *Principles of measurement systems* (4th ed.). Pearson Education.

[CR5] Büchau, Y.G., van Kesteren, B., Platis, A., Bange, J. (2022). An Autarkic Wireless Sensor Network to Monitor Atmospheric CO2 Concentrations. *Meteorologische Zeitschrift (Contrib. Atm. Sci.)*, 10.1127/metz/2022/1125

[CR6] Büchau, Y., Dörner, L., Bange, J. (2023). Short-term Comprehensive CO2 Degassing Dataset from a Mofette at the Starzach Site in Winter 2022 Obtained with a Custom Flow Meter, Including Atmospheric Variables [data set]. 10.1594/PANGAEA.963786. PANGAEA. Retrieved from https://doi.pangaea.de/10.1594/PANGAEA.963786 (https://doi.pangaea.de/10.1594/PANGAEA.963786).

[CR7] Burton, M. R., Sawyer, G. M., & Granieri, D. (2013). Deep Carbon Emissions from Volcanoes. *Reviews in Mineralogy and Geochemistry,**75*(1), 323–354. 10.2138/rmg.2013.75.11. https://pubs.geoscienceworld.org/msa/rimg/article-pdf/75/1/323/2954430/323_REV075C11.pdf

[CR8] Busch, N. E., & Kristensen, L. (1976). Cup anemometer overspeeding. *J. Appl. Meteorol.,**15*(12), 1328–1332. 10.1175/1520-0450(1976)015<1328:CAO>2.0.CO;2

[CR9] Camarda, M., De Gregorio, S., Capasso, G., Di Martino, R. M., Gurrieri, S., & Prano, V. (2019). The monitoring of natural soil CO2 emissions: Issues and perspectives. *Earth-Science Reviews,**198*, 102928. 10.1016/j.earscirev.2019.102928 Retrieved from https://www.sciencedirect.com/science/article/pii/S0012825219301151

[CR10] Camuffo, D. (2019). Measuring wind and indoor air motions. *Microclimate for cultural heritage* (pp. 483–511). Elsevier.

[CR11] Carapezza, M. L., & Granieri, D. (2004). CO2 soil flux at Vulcano (Italy): Comparison between active and passive methods. *Applied Geochemistry,**19*(1), 73–88. 10.1016/S0883-2927(03)00111-2 Retrieved from https://www.sciencedirect.com/science/article/pii/S0883292703001112

[CR12] Chevallier, F., Fisher, M., Peylin, P., Serrar, S., Bousquet, P., Bréon, F.-M, ..., Ciais, P. (2005). Inferring CO2 sources and sinks from satellite observations: Method and application to TOVS data. *Journal of Geophysical Research: Atmospheres*, *110*(D24), 10.1029/2005JD006390

[CR13] Chiodini, G., Cardellini, C., Amato, A., Boschi, E., Caliro, S., Frondini, F., Ventura, G. (2004). Carbon dioxide Earth degassing and seismogenesis in central and southern Italy. *Geophysical Research Letters*, *31*(7), 10.1029/2004gl019480

[CR14] Chiodini, G., Cioni, R., Guidi, M., Raco, B., & Marini, L. (1998). Soil CO2 flux measurements in volcanic and geothermal areas. *Applied Geochemistry,**13*(5), 543–552. 10.1016/S0883-2927(97)00076-0 Retrieved from https://www.sciencedirect.com/science/article/pii/S0883292797000760

[CR15] Dasgupta, R. (2013). Ingassing, Storage, and Outgassing of Terrestrial Carbon through Geologic Time. *Reviews in Mineralogy and Geochemistry.,**75*(1), 183–229. 10.2138/rmg.2013.75.7https://pubs.geoscienceworld.org/msa/rimg/article-pdf/75/1/183/2952840/183_REV075C07.pdf

[CR16] Elias, T., Sutton, A. J., Oppenheimer, C., Horton, K. A., Garbeil, H., Tsanev, V., ..., & Williams-Jones, G. (2006). Comparison of COSPEC and two miniature ultraviolet spectrometer systems for SO2 measurements using scattered sunlight. *Bulletin of Volcanology,**68*(4), 313–322. 10.1007/s00445-005-0026-5

[CR17] Etling, D. (2008). *Theoretische Meteorologie: Eine Einführung*. Springer-Verlag.

[CR18] Farrar, C. D., Sorey, M. L., Evans, W. C., Howle, J. F., Kerr, B. D., Kennedy, B. M., ..., & Southon, J. R. (1995). Forest-killing diffuse CO2 emission at Mammoth Mountain as a sign of magmatic unrest. *Nature,**376*(6542), 675–678. 10.1038/376675a0

[CR19] Feitz, A., Radke, B., Ricard, L., Glubokovskikh, S., Kalinowski, A., Wang, L., ..., & Credoz, A. (2022). The CO2CRC Otway shallow CO2 controlled release experiment: Fault characterization and geophysical monitoring design. *Int. J. Greenh. Gas Con.,**118*, 103667. 10.1016/j.ijggc.2022.103667

[CR20] Feitz, A., Schroder, I., Phillips, F., Coates, T., Negandhi, K., Day, S., ..., & Griffith, D. (2018). The Ginninderra CH4 and CO2 release experiment: An evaluation of gas detection and quantification techniques. *International Journal of Greenhouse Gas Control,**70*, 202–224. 10.1016/j.ijggc.2017.11.018

[CR21] Flohr, A., Schaap, A., Achterberg, E. P., Alendal, G., Arundell, M., Berndt, C., ..., & Connelly, D. (2021). Towards improved monitoring of offshore carbon storage: A real-world field experiment detecting a controlled sub-seafloor CO2 release. *International Journal of Greenhouse Gas Control,**106*, 103237. 10.1016/j.ijggc.2020.103237 Retrieved from https://www.sciencedirect.com/science/article/pii/S1750583620306629

[CR22] Foken, T. (2021). *Springer Handbook of Atmospheric Measurements*. Springer International Publishing. 10.1007/978-3-030-52171-4

[CR23] Forde, O. N., Cahill, A. G., Beckie, R. D., & Mayer, K. U. (2019). Barometric-pumping controls fugitive gas emissions from a vadose zone natural gas release. *Scientific Reports,**9*(1), 1–9. 10.1038/s41598-019-50426-3 31575969 10.1038/s41598-019-50426-3 PMC6773692

[CR24] Forster, P., Storelvmo, T., Armour, K., Collins, W., Dufresne, J.-L., Frame, D., . . . Zhang, H. (2021). The Earth’s Energy Budget, Climate Feedbacks, and Climate Sensitivity [Book Section]. In V. Masson-Delmotte et al. (Eds.), Climate change 021: The physical science basis. contribution of working group i to the sixth assessment report of the intergovernmental panel on climate change (pp. 923–1054). Cambridge, United Kingdom and New York, USA: Cambridge University Press. 10.1017/9781009157896.009

[CR25] Galle, B., Oppenheimer, C., Geyer, A., McGonigle, A. J., Edmonds, M., & Horrocks, L. (2003). A miniaturised ultraviolet spectrometer for remote sensing of SO2 fluxes: A new tool for volcano surveillance. *Journal of Volcanology and Geothermal Research,**119*(1), 241–254. 10.1016/S0377-0273(02)00356-6 Retrieved from https://www.sciencedirect.com/science/article/pii/S0377027302003566

[CR26] Gasser, T., Guivarch, C., Tachiiri, K., Jones, C. D., & Ciais, P. (2015). Negative emissions physically needed to keep global warming below . *Nature Communications,**6*(1), 7958. 10.1038/ncomms8958 10.1038/ncomms8958 26237242

[CR27] Gaubert, B., Stephens, B. B., Basu, S., Chevallier, F., Deng, F., & Kort, E.A.,..., Yin, Y. (2019). Global atmospheric CO2 inverse models converging on neutral tropical land exchange, but disagreeing on fossil fuel and atmospheric growth rate. *Biogeosciences (Online),**16*, 117–134. 10.5194/bg-16-117-2019 Retrieved from https://api.semanticscholar.org/CorpusID:56227065 10.5194/bg-16-117-2019 PMC6839691 31708981

[CR28] Glennon, J. A., & Pfaff, R.M. (2005). The operation and geography of carbon dioxide-driven, cold-water “geysers”. *The GOSA Transactions*, *9*, 184–192, Retrieved from https://www.researchgate.net//profile/Alan-Glennon/publication/216876596_The_operation_and_geography_of_carbon-dioxide-driven_cold-water_geysers/links/5b444580458515f71cb8a698/The-operation-and-geography-of-carbon-dioxide-driven-cold-water-geysers.pdf

[CR29] Gurrieri, S., & Valenza, M. (1988). Gas transport in natural porous mediums: A method for measuring CO2 flows from the ground in volcanic and geothermal areas. *Rend. Soc. Ital. Mineral. Petrol.,**43*, 1151–1158.

[CR30] Han, W. S., Lu, M., McPherson, B. J., Keating, E. H., Moore, J., Park, E., ..., Jung, N.-H. (2013). Characteristics of CO2-driven cold-water geyser, Crystal Geyser in Utah: Experimental observation and mechanism analyses. *Geofluids,**13*(3), 283–297. 10.1111/gfl.12018

[CR31] Haro, K., Ouarma, I., Nana, B., Bere, A., Tubreoumya, G. C., Kam, S. Z., ..., Koulidiati, J. (2019). Assessment of CH4 and CO2 surface emissions from Polesgo’s landfill (Ouagadougou, Burkina Faso) based on static chamber method. *Adv. Clim. Change Res.,**10*(3), 181–191. 10.1016/j.accre.2019.09.002 Retrieved from https://doi.org/www.sciencedirect.com/science/article/pii/S1674927819300929

[CR32] Holloway, S., Pearce, J., Hards, V., Ohsumi, T., & Gale, J. (2007). Natural emissions of CO2 from the geosphere and their bearing on the geological storage of carbon dioxide. *Energy,**32*(7), 1194–1201. 10.1016/j.energy.2006.09.001 10.1016/j.energy.2006.09.001

[CR33] Horton, K. A., Williams-Jones, G., Garbeil, H., Elias, T., Sutton, A. J., Mouginis-Mark, P., ..., Clegg, S. (2006). Real-time measurement of volcanic SO2 emissions: Validation of a new UV correlation spectrometer (FLYSPEC). *B. Volcanol.,**68*(4), 323–327. 10.1007/s00445-005-0014-9

[CR34] Inguaggiato, S., Vita, F., Cangemi, M., Calderone, L. (2020). Changes in CO2 soil degassing style as a possible precursor to volcanic activity: The 2019 case of stromboli paroxysmal eruptions. *Applied Sciences,**10*(14), 10.3390/app10144757 Retrieved from https://www.mdpi.com/2076-3417/10/14/4757

[CR35] IPCC. (2006). Guidelines for National Greenhouse Gas Inventories (H. Eggleston, L. Buendia, K. Miwa, T. Ngara, & K. Tanabe, Eds.). Inst. Glob. Environ. Strat. Retrieved from https://www.ipcc-nggip.iges.or.jp/public/2006gl/index.html (Prepared by the National Greenhouse Gas Inventories Programme)

[CR36] Jonas, M., Bun, R., Nahorski, Z., Marland, G., Gusti, M., & Danylo, O. (2019). Quantifying greenhouse gas emissions. *Mitigation and Adaptation Strategies for Global Change,**24*(6), 839–852. 10.1007/s11027-019-09867-4 10.1007/s11027-019-09867-4

[CR37] Jones, D., Barkwith, A., Hannis, S., Lister, T., Gal, F., Graziani, S., ..., Widory, D. (2014). Monitoring of near surface gas seepage from a shallow injection experiment at the CO2 Field Lab. *Norway. Int. J. Greenh. Gas Con.,**28*, 300–317. 10.1016/j.ijggc.2014.06.021

[CR38] Jung, N.-H., Han, W. S., Han, K., & Park, E. (2015). Regional-scale advective, diffusive, and eruptive dynamics of CO2 and brine leakage through faults and wellbores. *Journal of Geophysical Research: Solid Earth,**120*(5), 3003–3025. 10.1002/2014jb011722 10.1002/2014jb011722

[CR39] Kerrick, D. M. (2001). Present and past nonanthropogenic CO2 degassing from the solid earth. *Reviews of Geophysics,**39*(4), 565–585. 10.1029/2001RG000105 10.1029/2001RG000105

[CR40] Lowenstern, J. B. (2001). Carbon Dioxide in Magmas and Implications for Hydrothermal Systems. *Mineralium Deposita,**36*(6), 490–502. 10.1007/s001260100185 10.1007/s001260100185

[CR41] Lübben, A., & Leven, C. (2018). The Starzach site in Southern Germany: a site with naturally occurring CO2 emissions recovering from century-long gas mining as a natural analog for a leaking CCS reservoir. *Environ. Earth Sci.*, *77*(316), 10.1007/s12665-018-7499-y

[CR42] Lübben, A., & Leven, C. (2022). A gas-flow funnel system to quantify advective gas emission rates from the subsurface. *Environmental Earth Sciences,**81*(15), 1–11. 10.1007/s12665-022-10512-836760368 10.1007/s12665-022-10512-8

[CR43] Mauder, M., Foken, T., Aubinet, M., & Ibrom, A. (2021). Eddy-Covariance Measurements. In T. Foken (Ed.), *Springer handbook of atmospheric measurements* (pp. 1485–1515). Cham: Springer International Publishing. 10.1007/978-3-030-52171-4_55

[CR44] McGonigle, A. J. S., Oppenheimer, C., Galle, B., Mather, T. A., & Pyle, D.M. (2002). Walking traverse and scanning DOAS measurements of volcanic gas emission rates. *Geophysical Research Letters*, *29*(20), 46–1–46–4, 10.1029/2002GL015827

[CR45] Müller, M., Graf, P., Meyer, J., Pentina, A., Brunner, D., Perez-Cruz, F., & Emmenegger, L. (2020). Integration and calibration of non-dispersive infrared (NDIR) CO2 low-cost sensors and their operation in a sensor network covering Switzerland. *Atmospheric Measurement Techniques,**13*(7), 3815–3834. 10.5194/amt-13-3815-2020 Retrieved from https://amt.copernicus.org/articles/13/3815/2020

[CR46] Nilson, R. H., Peterson, E. W., Lie, K. H., Burkhard, N. R., & Hearst, J. R. (1991). Atmospheric pumping: A mechanism causing vertical transport of contaminated gases through fractured permeable media. *J. Geophys. Res. - Sol. Ea.,**96*(B13), 21933–21948. 10.1029/91JB01836 10.1029/91JB01836

[CR47] OpenStreetMap contributors (2023). *Planet dump retrieved from*[SPACE]https://planet.osm.org. https://www.openstreetmap.org.

[CR48] Pan, G., Xu, Y., & Ma, J. (2021). The potential of CO2 satellite monitoring for climate governance: A review. *Journal of Environmental Management,**277*, 111423. 10.1016/j.jenvman.2020.111423 Retrieved from https://www.sciencedirect.com/science/article/pii/S0301479720313487 10.1016/j.jenvman.2020.111423 33031999

[CR49] Papadopoulos, K. H., Stefantos, N. C., Paulsen, U. S., & Morfiadakis, E. (2001). Effects of turbulence and flow inclination on the performance of cup anemometers in the field. *Boundary-Layer Meteorology,**101*(1), 77–107. 10.1023/A:1019254020039 10.1023/A:1019254020039

[CR50] Pérez, N. M., Melián, G. V., Hernández, P. A., Padrón, E., Padilla, G. D., Baldago, M. C., ..., Lagmay, A. M. (2022). Diffuse CO2 degassing precursors of the January 2020 eruption of Taal volcano. *Philippines. Sci. Rep.,**12*(1), 19091. 10.1038/s41598-022-22066-7 10.1038/s41598-022-22066-7 PMC9646843 36351952

[CR51] Pickett-Heaps, C. A., Rayner, P. J., Law, R. M., Ciais, P., Patra, P. K., Bousquet, P., ..., Sweeney, C. (2011). Atmospheric CO2 inversion validation using vertical profile measurements: Analysis of four independent inversion models. *Journal of Geophysical Research: Atmospheres,**116*(D12), D12305. 10.1029/2010JD014887

[CR52] Privat, R., & Jaubert, J.-N. (2014). Predicting the Phase Equilibria of Carbon Dioxide Containing Mixtures Involved in CCS Processes Using the PPR78 Model. C.R.V. do Morgado and V.P.P. Esteves (Eds.), *CO2 Sequestration and Valorization* (chap. 15). Rijeka: IntechOpen. 10.5772/57058

[CR53] Rinehart, J. S. (1972). Fluctuations in geyser activity caused by variations in Earth tidal forces, barometric pressure, and tectonic stresses. *Journal of Geophysical Research,**77*(2), 342–350. 10.1029/JB077i002p00342 10.1029/JB077i002p00342

[CR54] Rogie, J. D., Kerrick, D. M., Chiodini, G., & Frondini, F. (2000). Flux measurements of nonvolcanic CO2 emission from some vents in Central Italy. *J. Geophys. Res.,**105*(B4), 8435–8445. 10.1029/1999JB900430 10.1029/1999JB900430

[CR55] Schäfer, M., Richter, M., & Span, R. (2015). Measurements of the viscosity of carbon dioxide at temperatures from (253.15 to 473.15)K with pressures up to 1.2MPa. *J. Chem. Thermodyn.,**89*, 7–15. 10.1016/j.jct.2015.04.015 Retrieved from https://www.sciencedirect.com/science/article/pii/S002196141500107X

[CR56] Scholz, K., Ejarque, E., Hammerle, A., Kainz, M., Schelker, J., Wohlfahrt, G. (2021). Atmospheric CO2 exchange of a small mountain lake: limitations of eddy covariance and boundary layer modeling methods in complex terrain. *J. Geophys. Res. – Biogeo.*, *126*, e2021JG006286, 10.1029/2021JG006286

[CR57] Štigler, J. (2012). Analytical Velocity Profile in Tube for Laminar and Turbulent Flow. Retrieved from Retrieved from 10.13140/2.1.3153.5046

[CR58] UNFCCC. (2015). Adoption of the Paris Agreement. https://unfccc.int/process-and-meetings/the-paris-agreement. Retrieved from https://unfccc.int/process-and-meetings/the-paris-agreement; https://unfccc.int/resource/docs/2015/cop21/eng/l09r01.pdf (United Nations Framework Convention on Climate Change)

[CR59] Wallace, J.M., & Hobbs, P.V. (2006). Atmospheric Science: An Introductory Survey (2nd ed., vol. 92). Elsevier. ISBN 9780127329512

[CR60] Werner, C., & Cardellini, C. (2006). Comparison of carbon dioxide emissions with fluid upflow, chemistry, and geologic structures at the Rotorua geothermal system. *New Zealand. Geothermics,**35*(3), 221–238. 10.1016/j.geothermics.2006.02.006 Retrieved from https://www.sciencedirect.com/science/article/pii/S0375650506000186

[CR61] Werner, C., Fischer, T.P., Aiuppa, A., Edmonds, M., Cardellini, C., Carn, S., ..., et al. (2019). Carbon Dioxide Emissions from Subaerial Volcanic Regions: Two Decades in Review. In B.N. Orcutt, I. Daniel, & R. Dasgupta (Eds.), Deep carbon: Past to present (pp. 188–236). Cambridge University Press. Retrieved from https://www.cambridge.org/core/books/deepcarbon/carbon-dioxide-emissions-from-subaerial-volcanicregions/F8B4EFAE0DAF5306A8D397C23BF3F0D7

[CR62] Williams-Jones, G., Stix, J., Hickson, C. (2008). *The COSPEC Cookbook: Making SO2 Measurements at Active Volcanoes*. IAVCEI, Methods in Volcanology. 10.13140/RG.2.2.13728.99845

[CR63] Winson, A. E. G., Costa, F., Newhall, C. G., & Woo, G. (2014). An Analysis of the Issuance of Volcanic Alert Levels During Volcanic Crises. *Journal of Applied Volcanology,**3*(1), 14. 10.1186/s13617-014-0014-6 10.1186/s13617-014-0014-6

[CR64] Woith, H., Vlček, J., Vylita, T., Dahm, T., Fischer, T., Daskalopoulou, K, ..., Lanzendörfer, M. (2023). Effect of Pressure Perturbations on CO2 Degassing in a Mofette System: The Case of Hartoušov, Czech Republic. *Geosciences*, *13*(1), 10.3390/geosciences13010002 Retrieved from https://www.mdpi.com/2076-3263/13/1/2

[CR65] Zhao, J., Zhang, M., Xiao, W., Wang, W., Zhang, Z., Yu, Z., ..., Lee, X. (2019). An evaluation of the flux-gradient and the eddy covariance method to measure CH4, CO2, and H2O fluxes from small ponds. *Agricultural and Forest Meteorology,**275*, 255–264. 10.1016/j.agrformet.2019.05.032

